# Therapeutic strategy regarding suppuration as a complication of abdominal wall defect repair with prosthetic material

**DOI:** 10.1186/1471-2334-14-S7-P76

**Published:** 2014-10-15

**Authors:** Răzvan Vasile Stoian, Daniel Ion, Simona Elena Albu, Andreea Teodora Topor, Dan Nicolae Păduraru

**Affiliations:** 1 University Emergency Hospital of Bucharest, Romania

## Background

Abdominal wall defects repair using prosthetic material is currently considered as the best available solution for most incisional hernias. Postoperatory suppuration remains the most frequent and fearful complication after this kind of intervention therefore imposing prevention methods. The aim of our study was to evaluate this serious yet controllable complication in the situation of a known pathogenic agent.

## Case series

This retrospective case series was carried on in the Surgery and Emergency Clinic III of the University Emergency Hospital Bucharest. It includes 14 cases of suppuration after abdominal wall reconstruction using prosthetic material during the last 15 years.

## Conclusion

In the situation of specific pathogenic agent, the use of specific antibiotic therapy and certain rather simple surgical techniques could guarantee therapeutic success for the doctor as well as the patient. A very good collaboration between the surgeon and the infectious disease physician was the key to therapeutic success.

